# Experiences of family members when a parent is hospitalized for their mental illness: a qualitative systematic review

**DOI:** 10.1186/s12888-023-04530-4

**Published:** 2023-01-20

**Authors:** Andrea Reupert, Phillip Tchernegovski, Lingling Chen, Maddison Huddle

**Affiliations:** School of Educational Psychology and Counselling, Faculty of Education, Monash University, Clayton, VIC 3800 Australia

**Keywords:** Psychiatric unit, Inpatient, Parents, Families, Children, Family-focused care

## Abstract

**Background:**

A considerable proportion of people attending mental health services are parents with dependent children. Parental mental illness can be challenging for all family members including the parent’s children and partner. The hospitalization of the parent and subsequent separation from dependent children may be a particularly challenging time for all family members. The aim of this paper was to review qualitative studies of family members’ experiences when parents, who have dependent children, were hospitalized for their mental illness. The experiences of parents themselves, their children aged 0–18 (including retrospective accounts of adults describing their childhoods), and other family members are included.

**Methods:**

This systematic review followed Cochrane Collaboration and PRISMA guidelines. A search was performed with keywords relating to parents, mental illness, psychiatric treatment, inpatient units, family members and experiences. Databases included CINAHL Plus, PsycINFO, ProQuest, MEDLINE, PubMed and Scopus. Quality assessment was undertaken using an expanded version of the Critical Appraisal Skills Programme. Thematic synthesis was conducted on the included papers.

**Results:**

Eight papers were identified. The quality assessment was rated as high in some papers, in terms of the clarity of research aims, justification of the methodology employed, recruitment strategy and consideration of ethics. In others, the study design, inclusion criteria and reporting of participant demographics were unclear. Family experiences of pressure and additional responsibilities associated with the parent receiving inpatient treatment were identified along with the family’s need for psychoeducational information, and guidance when visiting the parent in hospital. Children expressed various emotions and the need to connect with others. The final theme related to adverse impacts on the parent–child bond when the parent was hospitalized.

**Conclusion:**

The limited research in this area indicates that the needs of families are not being met when a parent is hospitalized for their mental illness. There is a considerable need for adequate models of care, family-focused training for staff, and psychoeducational resources for families. Additional research in this area is essential to understand the experiences of different family members during this vulnerable time.

## Background

Families where a parent has a mental illness and is caring for dependent children, are some of the vulnerable in our communities. Between 20 and 38% of clients who attend adult mental health services are parents caring for children below the age of 18 [1]. Children who grow up with a parent with a severe and chronic mental illness are at substantial risk of acquiring their own difficulties with mental health and/or substance abuse [2]. Other potential adverse outcomes include an increased risk of injury, poor school readiness, and stress-related somatic health conditions such as asthma [3–5]. Partners of those who have a mental illness reported feelings of loss and isolation, and described themselves as more of a caregiver than a partner [6]. Likewise, grandparents reported assuming major caring responsibilities for dependent children when a parent has a mental illness, especially when hospitalized [7]. Without targeted support for their parenting role, some parents may have their parenting compromised [8] and lose custody of their children [9], which in turn impacts negatively on their mental health and recovery [10].

The needs of children and other family members in families where parents have a mental illness are not always identified by mental health clinicians [11]. Due to funding requirements and deficits in clinicians’ knowledge and skill [12], parents’ treatment is often prioritized over the needs of children, unless there are issues with neglect, abuse or the child presents with their own psychological issues [13]. The needs of the parent’s partner and other extended families is likewise not routinely considered by clinicians, resulting in isolation, their own mental health challenges and relationship strains [6]. However, adverse child and other family members’ outcomes are not inevitable. Emerging results highlight the efficacy of manualized interventions for these families [14]. Instead, or in addition to standard care, organizations can offer a whole family approach, across screening, intake, treatment planning and delivery [15]. Such guidelines see parenting status recorded at intake, targeted support provided for the client’s parenting role, and appropriate interventions and supports offered to children and other family members.

Family focused practice (FFP) is a term that is often used to describe the way in which clinicians might engage with parents, their partners, children and other family members. FFP extends the focus of care beyond the parent’s mental health to assess and respond to the wellbeing of all family members, including children, while also acknowledging and supporting a client’s parenting role [16]. Acknowledging and celebrating a client’s parenting status can be instrumental in a client’s recovery journey by offering hope and connectedness, as well as honoring their identity as a parent [10]. It also facilitates parental confidence and competence to nurture their children, within the context of their illness [17].

Notwithstanding the prevalence and needs of these families, and the utility of a family focused approach, there is evidence that adult mental health services do not adequately respond to the clients who are parents caring for dependent children. Audits of clinicians’ case notes indicate that FFP is not commonly employed in adult mental health settings in the UK, New Zealand and the USA [18–20]. Some clinicians do not believe that it is appropriate, nor within their remit, to support clients’ children [21]. Even when issues relating to parenting responsibilities and child wellbeing are considered within their role, adult mental health clinicians have indicated a lack of training that might address deficits in their skill and knowledge [12]. Similarly, others have found that clinicians rarely have the time nor the skills to engage with partners of those with mental health challenges [6].

Additionally, services may not consistently identify nor respond to the needs of families when a parent is hospitalized for their mental illness. Previous research has predominately investigated this field in relation to nurses’ experiences of children visiting their parents in psychiatric facilities. Korhonen et al. [22] found that nurses rarely met with the children of clients, while O’Brien et al. [23] found that nurses were unsure of their role and not sure what they might say to clients’ children. In a review of studies that identified the practices of mental health nurses in psychiatric facilities, Foster et al. [24] found that there were logistical issues for children visiting units, parenting status was not identified at intake, and an overall lack of organizational support for FFP. The experiences of other family members, such as partners and grandparents, when the parent is hospitalized, is less clear.

The hospitalization, and often unexpected separation of parents from their families, can be confusing and distressing for the parent, children and other family members [25]. Knowing more about the experiences of family members when a parent is hospitalized can be used to inform service planning and delivery. Qualitative methods allow for in-depth, contextualized analysis and are particularly relevant to the examination of lived experiences of those impacted by mental illness. To facilitate the translation of the extant body of available qualitative research, a systematic synthesis of the research is required. The aim of this review is to identify the experiences of children under the age of 18 (including adult children’s retrospective accounts of their childhood), the parent, their partner and other family members, when a parent is hospitalized for their mental illness. The research questions were (i) what is the nature of existing qualitative research investigating the experiences of families when a parent is hospitalized for their mental illness? And, (ii) What does this research tell us about these experiences of different family members during this time, including the parent, children, partner, grandparents and other family members? Such information might be used to guide policy, and inform professional development training and practice guidelines when working with families.

## Methods

The focus of the review was the experiences of parents and other family members (e.g., children aged 0–18 years, partner, grandparents, and other extended family members) when a parent is hospitalized for their mental illness. For the purposes of this review, the concepts of parent and family were defined broadly. Parenthood and family were not restricted to biological relationships or living arrangements. Studies relating to biologically unrelated and non-custodial parents were considered relevant, as long as the inpatient had some ongoing parental involvement. Similarly, it was recognized that the concept of family may vary across cultures, circumstances, and time periods [26]. We utilize the definition provided by Osher and Osher [27], where family is “defined by its members, and each family defines itself.” Therefore, no restrictions were imposed on the types of families in this review.

### Design

This systematic review employed the Cochrane Collaboration method [28, 29] and the Preferred Reporting Items for Systematic Reviews and Meta-Analyses (PRISMA) model [30] for searching and retrieving eligible studies.

### Search strategy

Preliminary searches were employed to determine the quantity and nature of existing research relevant to the review and to trial potential search terms. Searches were conducted across six databases: CINAHL Plus, PsycINFO, ProQuest, MEDLINE, PubMed, and Scopus. Relevant content areas for the review were identified and used to generate subject headings and search terms. These were refined through several preliminary searches to promote maximum inclusiveness for the review. The headings and terms utilized in this review are presented in Table 1. Where databases provided the option, search terms were mapped to subject headings to increase the records identified by the search. Filters were used where search platforms allowed, including peer-reviewed, English, and full-text. The search strategy was developed in consultation with a university librarian. Two searches were conducted; the first search captured papers published between 2004 and 30th October 2020 when this review was initiated and the second for papers published between 30th October 2020 and 13th January 2022 to provide an update of the review and ensure results were as recent as possible. Results were revised (and not only added to) in light of the two new papers identified. This date range was employed to capture research of key pioneers within the field yet ensured the scope of research addressed current practices within psychiatric units in recent times.

**Table 1:** Search terms used within the databases to find relevant papers

Content	Search Terms
Parent	parent* OR mother* OR father* OR spouse*
	AND
Mental illness	“mental illness*” OR “mental disorder*” OR “mental disabilit*” OR “psychotic disorder*” OR anxiety OR depression OR schizophrenia OR “personality disorder*” OR “severe mental illness*” OR “chronic mental illness*” OR “trauma-related disorder*” OR COPMI OR FAPMI
	AND
Psychiatric unit	“psychiatric unit*” OR “psychiatric ward*” OR “psychiatric service*” OR “psychiatric hospital*” OR “mental health ward*” OR “mental health service*”
	AND
Inpatient treatment	admission OR inpatient* OR consumer* OR client* OR hospitali*ation OR treatment OR “family-focused care”
	AND
Family member	“family member*” OR famil* OR child* OR mother* OR father* OR parent* OR spouse* OR husband* OR wife* OR grandparent* OR grandmother* OR grandfather* OR relative*
	AND
Experience	experience* OR “life experience*” OR “childhood experience*” OR parenthood

### Study selection

The first search resulted in 1328 records. Duplicates were removed, leaving 830 records. These were screened in accordance with the inclusion/exclusion criteria. Inclusion criteria included qualitative papers that explicitly stated the parent had been hospitalized for psychiatric treatment and described the experiences of the parent or other family members during this period. Studies of adult participants providing a retrospective account of their childhood were included. Those studies that outlined the experiences of parents who had been hospitalized were also included, if the focus was on their experiences as a parent not generally on their experiences of being a patient of a psychiatric unit. Exclusion criteria included those studies that explored family members’ experiences of parental mental illness, rather than how the hospitalization impacted family members. Studies were removed if they investigated the experiences of mental health clinicians as were those that focused on service provision for child psychopathology, specialized settings, such as mother-baby units, or specific interventions such as parenting interventions. Given the different treatment needs of parents who experience substance misuse issues [31, 32], studies with those parents were excluded. Studies were omitted if they did not present primary qualitative data. The removal of duplicates and initial screening was completed with the RAYYAN online software [33]. As papers were screened, the reasons for exclusion were recorded and tracked by the software.

One author screened all 830 records by title and abstract. For instances where abstracts were missing from the record, they were obtained through online searches. The first and last 110 records (26%) were independently screened by two other authors. A blind setting was utilized on the RAYYAN software to ensure independent screening decisions between authors. An inter-rater discrepancy occurred for 12 (5%) of the double-screened papers. All three researchers discussed these discrepancies in relation to the inclusion/exclusion criteria until a consensus was reached. This initial screening of titles and abstracts resulted in 51 remaining entries. The reference lists of all identified papers as well as relevant reviews were hand searched, resulting in the inclusion of one additional paper.

**Fig. 1:**
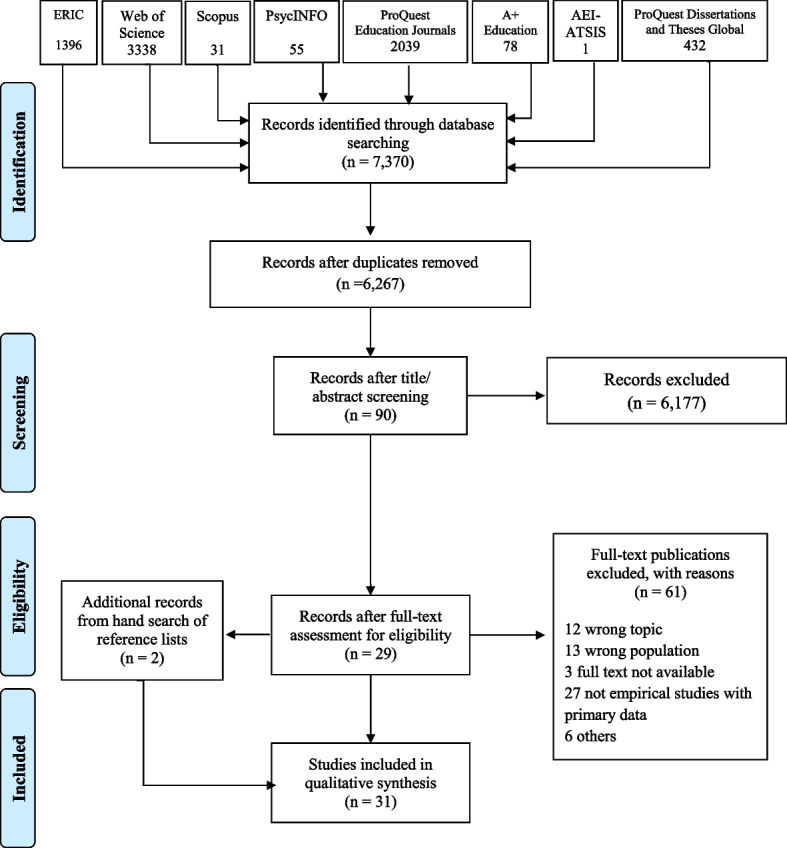
PRISMA flow diagram

Full-text screening was conducted on the 52 identified studies independently by two of the authors. As with the initial screening, reasons for studies being excluded at this stage were recorded. Discussions were undertaken as necessary to clarify exclusion/inclusion criteria. The main reasons for excluding studies at this stage were because they focused on settings other than psychiatric inpatient units or investigated the inpatient treatment of people who were not parents. One study with a central focus on substance abuse disorders was also excluded. Studies with mixed methods were removed if qualitative data were not developed into themes. Six papers remained after this full-text round of screening. Following an updated search and screening on 13th January 2022, two additional studies were identified, resulting in a total of eight studies for final inclusion in this review. A PRISMA diagram of the full search and screening processes is shown in Fig. 1.

### Quality assessment

A quality assessment tool, based on the Critical Appraisal Skills Programme [34], was employed to assess the quality of included papers. It evaluated the study context (appropriate research design to address aims; identification of inclusion/exclusion criteria), quality of analytical methods (inter-rater reliability; multiple analysis to demonstrate the rigor of research), and the involvement of relevant parties during the study (development of interview schedules; member checks). The quality of papers was assessed by rating whether the study met each criterion fully (2 points), partially (1 point), or not at all (0 points). This process provided a quality rating for each paper ranging from 0 (poor quality) to 62 (high quality). Quality assessments were completed by two of the authors for each paper. Discrepancies were small and ranged between 0–3 points (0–5% of the total score), with the mean of the authors’ scores being calculated as the final assessment score. The final assessment ratings ranged between 18.5 indicating low quality [35] and 52.5 indicating high quality [36] with a mean of 44.9 (medium quality). Quality ratings for each paper are presented in Table 2. Given the limited number of studies identified, all papers were considered equally in the synthesis of results, regardless of their assessed quality. Studies rated highly had clear research aims, provided a sound justification for the methodology employed, considered the ethics involved in conducting the research and provided clearly articulated recruitment strategies appropriate to the aims of the study. Studies rated low were not clear in their research aims, nor how they justified the study design employed and the reporting of participants’ demographics and participants’ inclusion criteria.

**Table 2:** Characteristics of included papers

Authors (year)	Country	Participants	Study Design	Quality rating	Aim of study	Key qualitative findings related to the hospitalization of parents
Blegen et al. [37]	Norway	Mothers (*n* = 10) with children aged 0–18 years, admitted to a hospital unit; diagnoses included: depression, anxiety, bipolar, disorder, ADHD	Semi-structured interviews, analysis with philosophical hermeneutics	40.5	To understand the experience of being cared for in psychiatric care as a patient and as a parent	**Parental ambivalence**: Mothers’ anxiety about disclosing their inner feelings about themselves as a mother to clinicians despite wanting support in this area; a mask of silence provides protection but also causes distance and reduced opportunity for optimal care; struggle between responsibility and condemnation
Knutsson-Medin et al. [36]	Sweden	Adult children (*n* = 36): mean age of 25.8 years, range 19–38 years; 15 men, 21 women; parents had been previous psychiatric inpatients	Written survey, manifest content analysis	52.5	To examine adult children’s previous experiences of contact with their parent’s psychiatric inpatient services	**Children’s needs:** Support and information needed from hospital staff and more contact with clinicians **Pressure:** relief that the parent was being looked after **Involvement and guidance:** Children were not provided with information about their parent during hospitalization
Kosman et al. [35]	USA	One 28-year-old mother with postpartum depression and admitted to inpatient psychiatry (not a mother-baby unit)	Case study	18.5	To discuss the issues associated with postpartum depression including mother and infant safety, maternal-infant attachment, psychopharmacological options for postpartum depression, and traumatic birth experiences	**Parental ambivalence:** Parental ambivalence about hospitalization, experiences of emotional difficulties being separated from her infant, logistical and physiological issues associated with breastfeeding and challenges of coordinating childcare during hospitalization
Maybery et al. [38]	Australia	5 parents with mood disorder, 3 anxiety, 3 personality disorder, 1 psychotic (total *n* = 10), partners (*n* = 2); children (aged 6–16 years) (*n* = 12). Total *n* = 24	Focus groups, qualitative interpretive analysis Quantitative questionnaire, between groups analysis of variance	51.5	To examine child, parent and professional perspectives of the needs of children when a parent is an inpatient and how to best meet those needs	**Pressure:** Older children had to care for themselves while parent was in hospital **Involvement and guidance:** Families wanted information about the parent’s mental illness **Children’s needs:** Facilitate opportunities to see their parents; to be recognized/identified by professionals during admission and discharge; siblings are an important source of support
O'Brien et al. [25]	Australia	Parents (*n* = 5) discharged from an inpatient facility in the last 12 months; with a child aged 0–18 years, partners (*n* = 2), children (*n* = 5) aged between 8–15 years; and grandparents (*n* = 1). Total *n* = 13	Semi-structured interviews, thematic analysis	47	To examine perspectives of children, parents and carers towards children visiting parents in inpatient units	**Pressure:** Family stress when making decisions about children visiting their parent in hospital and caring for children during visits **Involvement and guidance:** Family involvement in admission interviews was appreciated; wanted guidance on child visitations with parents **Children’s needs:** Support during visits, including advice and debrief from staff **Parental ambivalence:** Parents wanting children to visit but also wanting to shield them
Skundberg-Kletthagen et al. [39]	Norway	Partners (*n* = 6), grandparents (*n* = 2), siblings (*n* = 2), children aged over 18 years (*n* = 12) and participants who had “other relationships” (*n* = 2). The parent had been admitted to a psychiatric ward with depression. Total *n* = 24	Semi-structured interviews, interpretive phenomenological analysis	51.5	To describe experiences of encountering a psychiatric service as a relative of an inpatient with severe depression	**Pressure**: Difficulty getting parents admitted; burden of care after discharge **Involvement and guidance:** Families wanted more information about psychiatric services and to be included in decision making, particularly around discharge planning
Wells et al. [40]	UK	Fathers (*n* = 8) aged 27–54 years (M = 43 years), admitted in forensic inpatient care with current admissions ranging from 3 months to 9 years; having children aged 2 to 39 years	Semi-structured interviews, grounded theory	46.5	To investigate men’s experience of fatherhood in forensic inpatient care	**Parental ambivalence:** Physical absence not necessarily leading to psychological disconnection; influential factors of psychological connection (e.g., geographical location of hospital admission and financial difficulties, the child’s mother, participants’ own parents, professional support); ways and challenges of fulfilling parenting responsibilities during hospitalization
Zeighami et al. [41]	Iran	Children (*n* = 10) aged between 17–26 years, daughter in law (*n* = 1), grandmothers (*n* = 2). Parents had mood disorder, schizophrenia, obsessive disorder and had been hospitalized for an average of 13 times. Total *n* = 13	Semi-structured interviews, grounded theory	51	To explore the mental health needs of children at various stages of their parent’s mental illness	**Pressure:** Burden on children to convince parents to seek support/admission to the psychiatric unit **Children’s needs:** People to be with and talk to during parent hospitalization; **Involvement and Guidance:** After discharge they need guidance and education about the situation

### Data extraction

An excel spreadsheet was established to extract data including the country in which the study was conducted, participant demographics, study design, quality rating, study aim/s, and qualitative findings related to family’s experiences of the hospitalization of parents. When recording results, both primary data (participants’ excerpts) as well as secondary data (authors’ interpretations of participants’ experiences) relevant to family experiences when a parent is hospitalized were reviewed, with notes made to distinguish the two.

### Data analysis

Thematic synthesis, an inductive approach that adapts a ‘critical realist’ approach [42], was undertaken to analyse results. Thematic synthesis was selected because it is an established, effective method for identifying, evaluating and reporting themes in systematic reviews [43], and is well suited to our objective of aggregating and distinguishing participants’ experience, as per Johns et al. [44]. Informed by the approach of Thomas and Harden [43], two members of the research team read and re-read all articles several times to become thoroughly familiar with the content. In this process, the results of each study were coded line-by-line, after which the second author produced an initial list of codes (see the last column in Table 2). The initial list of codes was then reviewed across papers by two team members and amended, refined and restructured by going back to each individual study, and subsequently categorized into “descriptive themes”. Analytical themes, that went beyond the reported data, were developed, by iterative rereading of the original data and descriptive themes, and discussion amongst the author team, resulting in the four overarching themes presented here. Commonalities amongst, and differences between, family members were highlighted as appropriate.

## Results

### Study characteristics

There were two papers each from Australia and Norway, and the other four were from Sweden, Iran, the USA, and the UK. Five studies investigated the experiences of the parent who received psychiatric inpatient treatment [25, 35, 37, 38, 40]. One presented the recollections of adult children of when their parent was hospitalized [36]. Three studies presented the experiences of children under 18 years of age [25, 38, 39] and one study presented the views of children aged 17–26 years [41]. Three included the views of grandparents (parents of the individual who had been hospitalized) [25, 39, 41]. Four studies included multiple family members [25, 38, 39, 41]. Two papers exclusively focused on mothers receiving inpatient treatment [35, 37], and one focused specifically on fathers in forensic inpatient care [40]. Four studies included families where a parent of any gender was admitted to a psychiatric unit [25, 36, 38, 41]. See Table 2 for further details. Please note that the final column in Table 2 presents the initial codes identified by the research team, during the early stages of the analytic process, not verbatim themes identified in the primary studies.

### Thematic synthesis

Four themes were identified: pressure and responsibility; a need for information and guidance; the emotional needs of children; and the parent–child bond during hospitalization.

#### Theme 1: pressure and responsibility

Six of the eight studies described pressures experienced by family members when a parent received inpatient treatment [25, 36, 38–41]. Seeking admission for the parent was challenging for families. According to Skundberg-Kletthagen et al. [39], “…they describe it as a battle to get help” (p. 119). One participant highlighted how difficult admission was even though the parent had previously engaged with the system: “It shouldn’t be so difficult to be admitted to a psychiatric ward. It’s the same old story every spring…” ([39] p. 119). Convincing the parent to seek help was difficult for some with one child saying, “I use thousands of tricks to take him to hospital” ([41] p. 99).

When parents were admitted, there were changes to children’s responsibilities. Knutsson-Medin et al. [36] found that adult children’s recollections from their childhood when they experienced “…feelings of relief when someone was responsible for their parent during his or her hospitalization” (p. 748). One participant in their study illustrated this by saying “I appreciated it when my mother was taken care of in hospital. I knew that she ate, took her medicine, slept well and felt better” (p. 748). Conversely, Skundberg-Kletthagen et al. [39] reported adult children’s concerns about the quality of care their parents were receiving: “…there were a number of temporary staff and many people to relate to, and the doctor was away for six weeks when dad was admitted” (p. 120). Simultaneously, the authors noted that children felt “more secure when they experience the health personnel are accessible and observe changes in the patient” (p. 120).

Other pressures and responsibilities were identified. Two studies [36, 38] found that some adolescent children did not have relatives or other adults to care for them and accordingly experienced financial difficulties and “were required to find their own accommodation” ([38] p. 5). O’Brien et al. [25] and Wells et al. [40] highlighted the inconvenient appointment times for families wishing to visit the parent. Relatives caring for children were burdened with making decisions about whether children should visit their parents, and felt caught between the respective needs of children and parents. Likewise, Wells et al. [40] found the responsibility fell to other family members (primarily the mother) to support and maintain the father-child relationship. Supervising young children on the ward was another responsibility that other family members found difficult, especially when expected to attending interviews with hospital staff. O’Brien et al. [25] reported: “Several parents and carers indicated that they had disagreements about children visiting, with the carer/parent not wanting to facilitate children visiting” (p. 140).

Despite the relief of seeing their parents, O’Brien et al. [25] found that children shouldered multiple responsibilities when visiting. Some children viewed these visits as a form of support directed to their parents rather than for their own benefit. One child (age not reported) commented, “seeing us would make him want to get better faster” (p. 140). This is a notion that was sometimes reinforced by other family members. “One child commented that his mother had said: ‘Don’t be too anxious about it... we are just there for Dad, so just be there for Dad’” (p. 140). One concern for children was that they would say or do something to upset their parents. One remembered thinking, “Maybe I shouldn’t have said that... maybe I have made her worse” ([25] p. 141).

Discharge was another stressful time and when children wanted to know “when are you coming home?” ([40] p. 17). Discharge was also challenging for families and children when they had to resume care for the parent before they were ready. Skundberg-Kletthagen et al. ([39] p. 120) reported, “the period of hospitalisation was too short … the treatment had not been completed and … the patient was not well enough to be discharged – all of which put a great burden on relatives: ‘We said she couldn’t just be discharged; she wasn’t capable of managing herself, but in fact that’s what they intended to do!’ ([adult] daughter).” When single parents were discharged, children felt pressured with one adult recalling a prior experience, “I had to take care of my mother myself” ([36] p. 748).

#### Theme 2: a need for information and guidance

Identified in seven studies were family members’ needs for information and guidance about how the experience of hospitalization [25, 35, 36, 38–41]. However, these needs were often unmet by hospital staff. Some family members believed that parent confidentiality was prioritized at the expense of them being involved in treatment planning or being informed about the parent’s progress [39]. Children felt especially excluded from information about their parent’s treatment. One child (age unreported) said, “You shouldn’t be left in the dark because you’re a kid and maybe they don’t give us credit... you can handle it. It is much more scary not knowing” ([25] p. 141). According to Knutsson-Medin et al. [36], “some children claimed that they were not given any information even after their parent attempted suicide” (p. 748).

O’Brien et al. [25] found that parents, children, and other relatives were all disappointed at the lack of information or guidance about children visiting their parents in the hospital. When bringing children to the hospital, family members wanted advice on planning the visit and preparing children. They also wanted “assistance with the hellos and the goodbyes” (p. 141). Children wanted information about how to behave on the ward. However, “there was little assistance available when children visited or when the decision about visiting needed to be made. One carer noted: ‘No one took an interest in whether the child came in or not... I cannot say there was one staff member that counselled us’” ([25] p. 141). Fathers wanted professionals to help them build their bonds with children during hospital visits as they did not feel confident about doing this on their own [40]. Drawing on a single case study, Kosman et al. ([35] p. 282) recommended that mothers with postpartum depression be provided with “frequent and extended supervised visitations with the newborn; offering lactation consultation and a private space to pump and store breastmilk”.

Parents who were inpatients wanted staff to provide their children and partners with psychoeducation about their mental illness [38]. Children also said that they wanted this information [25, 36, 41]. O’Brien et al. [25] found that: “Children (particularly older children), who had been part of family interviews while their parent was hospitalized, appreciated being included and gaining some understanding of their parent’s illness” (p. 141). Finally, some children wanted guidance about how to interact with their parents after discharge: “We need to know how to deal with him and how to treat him… They should educate us about how to treat him to prevent conflicts” ([41] p. 99).

#### Theme 3: children’s emotional needs

Children experienced a range of emotions when their parents were hospitalized. Some children became lethargic, depressed and withdrawn [38, 41] while others described experiences of anxiety “that thought [that the parent is not going to get well] will stay with you... you don’t think logically... you think with your emotions” ([25] p. 141). Being in the hospital setting was emotionally difficult for some children. Recalling her childhood experiences of visiting a parent in hospital, one adult said, “The gloomy atmosphere and furnishings frightened me…” ([36] p. 749).

In four studies [25, 36, 38, 41], children of various ages described a need for connection and “someone to talk things through with” ([38] p. 5). An older child noted, “I need someone to sit and talk with me, throw me in another mood, and avoid remembering my past” ([41] pp. 98–99). Children sought this support from various sources. Parents who were inpatients and children both recognized that siblings often supported each other [38]. Extended family members were also important. An adult child recalled, “…we go to my grandmother or uncle’s home to avoid being alone. Our morale is damaged seriously when we are alone. However, when somebody comes to our home, greets us, and consoles us, our morale is boosted considerably” ([41] p. 99). One child relied on his dog for comfort: “my dog is the closest thing I have to human contact for days when my mum is in hospital” ([38] p. 6).

Despite families wanting hospital staff to provide support for children, they reported “…a lack of support from, and almost no contact from the staff” ([36] p. 750). Parents and children both indicated that children should be debriefed by staff after parents were admitted [38]. Similarly, one child said that “someone should see that the child is okay before they leave” the hospital after visiting parents ([25] p. 141). Likewise, some children wanted their own professional support with one adult participant reflecting, “I wished that I could have seen a social worker or a psychologist, someone who could have asked me about my reactions…” ([36] p. 749). Another suggested, “our school welfare officer should have been informed” ([36] p. 749) so that they could receive support through their school.

#### Theme 4: the parent–child bond during hospitalization

Four papers [25, 35, 37, 40] presented findings related to experiences of the parent–child bond when the parent received inpatient treatment. Some parents felt ambivalent about psychiatric hospitalization, and where as one mother described, she benefited from hospitalization for her mental health [35] but at the same time, reported that bonding with her child and being involved in childcare and parenting was a challenge, an experience also reported by fathers admitted in forensic inpatient care [40].

Two studies found that some parents experienced a sense of disconnection from their children when hospitalized [35, 40]. Even though they remained in contact with their children through telephone calls or visitation, two papers, one study with mothers ([35] p. 280) and the other with fathers ([40] p. 14) described “missing out on” their children’s development, and subsequently felt guilty and remorseful. Some fathers felt they were no longer able to voice their opinion on childcare issues [40]. However, for other fathers, physical absence from their children did not necessarily lead to psychological disconnection, as one father reported, “my kids will always be around me …if not physically, spiritually they will always be around me” ([40] p. 11). This psychological connection can be maintained by “cognitively holding their child(ren) in mind” ([40] p. 15) or through child contact, which “takes away the... severity of having to serve a sentence as such in a mental hospital… I can pick up the phone any time and phone the children ([40] p. 19).

The parent–child bond was also impacted by the practicalities involved in organising visits from children, which were not easy to organise with issues around transport and cost [25, 40]. The social support network, particularly the child/ren’s other parent and grandparents, were other factors impacting children’s ability to visit. One mother admitted to inpatient treatment was able to maintain her bond with her baby as her partner and mother took turns taking the child to the hospital and bringing her photos of the baby [35]. Likewise, one father attempted to keep a harmonious relationship with their child/ren’s mother “so I can speak to them [children] when I need to” ([40] p. 13). Conversely, other fathers indicated that their ex-partners kept their children away from them when in hospital and where “it’s down to your other half and whether they want to bring them” ([40] p. 19).

Some parents were unsure whether their children should visit them. Some parents recognized the benefits of seeing their children, e.g., “having family come to see (me) is one of the best cures” ([25] p. 139) and where seeing children was “the only positive that’s going on in my life” ([40] p. 11). Family members also recognized the benefit of visits for the parent as they may “not have seen their kids for many weeks... and it’s quite distressing for them to be separated and not to be able to reassure their kids that they are okay” ([25] p. 140). However, some parents indicated: “I don’t want anyone to let her know that I go to this sort of hospital”, which the authors reported was due to the “fear that her child would tell people where her mother was, and thus attract stigmatizing responses herself” ([25] p. 140).

Many parents were concerned about their children visiting them in hospital and where some mothers “feared that the diagnosis would come to define them as mothers. They felt they were at risk of losing their children” ([37] p. 6). Consequently, they minimized their mental health difficulties when talking to clinicians even though they knew it undermined their own recovery; “I’m probably afraid of being labelled a lunatic and then they will take my children away…” ([37] p. 7). Likewise, one father, concerned about child protection procedures indicated that “I don’t feel pleased... they [staff] are taking the moment from the visit” ([40] p. 21). Conversely, some inpatient professionals facilitated parents’ bond with their children by prompting visits and asking, “‘when is your daughter picking you up?’... they [staff] always want to know what else she [daughter] is doing... my boys come in and they tease them a little bit… it’s good like... very supportive and very important to me” ([40] p. 14).

Although challenging, some parents attempted to fulfil their parenting responsibilities in the confines of the hospital. One mother with postpartum depression pumped breast milk so that her partner could feed their baby [35] while some fathers in forensic inpatient care “enact[ed] paternal parenting practices” including providing emotional support, guidance, and money to their children ([40] p. 18).

## Discussion

This review sought to identify reported experiences of parents, children and other family members when a parent is hospitalized for their mental illness. When hospitalised, some children reported feeling relieved that the parent was being looked after, though others experienced financial and accommodation challenges. Relatives caring for children were unsure whether or how children should visit their parent when in hospital. Children were emotionally impacted by being separated from their parent and by the atmosphere of the hospital setting, not sure of how or whether to support their parent. Family members highlighted a need for psychoeducational information, and guidance for children visiting the parent in hospital. Children expressed various emotions and the need to connect with others. Finally, being hospitalized impacted adversely on the parent–child bond.

Many of the experiences of family members resonate with other research outlining issues for individuals being hospitalized, in terms of obtaining appropriate and timely support [45] and problems when discharged [46]. A family lens, that the identified studies afforded, indicates that these issues are experienced by more than the inpatient and also impact children, the partner and other family members. For example, family members, especially children, wanted their relative to obtain timely support; they also were not sure how to support their parent/relative when they were discharged from hospital. Parents’ uncertainty about family visits, and children’s largely negative experience of visiting their parent in hospital is perhaps not surprising given clinicians’ unease about family focused work in these settings and a lack of organisation guidance [24].

The review found that the needs of children were not adequately considered by hospital staff when visiting and supporting their parents in hospital. Consequently, children experienced various emotions, including being depressed and anxious. They also needed someone to talk to, or debrief about the experience of visiting their parent, and to provide them with information about their parent’s illness and treatment as well as accessing professional support for themselves. Both children and other family members (the other parent and grandparents) wanted guidance around whether children should be visiting their parent, and if so, how to best manage the visit. Each family is unique, and accordingly, it is critical that these discussions are supported by clinicians to occur with the family unit, allowing for all family members to say what they want and need, allowing also for these needs to change over time.

Acknowledging and supporting a client’s parenting needs can be an important part of their recovery journey [10]. The results of this review support this association for those who are hospitalized, but simultaneously identified parents’ fear of associative stigma due to the child’s relationship with their parent along with a fear about their child’s possible negative, judgemental reaction that the parent is in that “sort of hospital” ([25] p. 140). These stigma experiences have been documented elsewhere [47] and resonate with the experiences of families in the reviewed studies. Parents also expressed a fear of losing their children due to their illness, a fear that in some countries is well founded. In the USA for example, Roscoe et al. [9] found that the odds of having children removed from parents, more than doubled among parents with mental illness. Efforts to de-stigmatize parental mental illness in the public sphere and to promote mental health literacy in families [48] might serve to normalize mental health experiences, especially hospitalization, and promote empathy and understanding, within and outside of the family unit.

This review focused on peer-reviewed publications written in English which may have excluded relevant findings in other languages or within grey literature such as government publications or student dissertations. Only eight studies were identified. The term “carer” was not used in the search strategy and if used, may have resulted in additional papers. The mean quality rating of the eight studies was 44.9 (out of 62), ranging from 18.5 (low) to 52.5 (high). Most issues of quality related to undefined research aims and unclear reporting of study designs, inclusion criteria, participant demographics (particularly diagnosis), and data collection methods. Numerous papers were also unclear about which participants communicated particular ideas and quotes within the study results. Such limitations made it difficult to differentiate the needs of family members and accordingly, further research is required to elicit the potentially different needs of family members. Similarly, the respective views of mothers and fathers, who have been hospitalized, need to be differentiated, given their different experiences of parenting with a mental illness [49]. Experiences and needs across children’s ages and developmental stages are a key consideration for further research. Cultural and geographical factors should also be considered in future research, including different health care systems, availability of care, and cultural perspectives of mental health treatment and parenting. Likewise, future studies might examine the separate role and needs of other family members, including partners and grandparents. None of the papers considered whether the parent was a voluntary or involuntary inpatient and this warrants further exploration. The experiences of families where a parent has a substance misuse issue and is hospitalized might be the focus of a future review.

The results of the review along with previous research [10, 15, 16] suggest the need for best practice guidelines for hospital management around family focused practice, including strategies for working with different family members before, during and after periods of hospitalization. Guidelines concerning family friendly visiting rooms and appointment times might also be required. How parents and children might maintain contact during this time needs to be explored, along with child friendly psychoeducational resources [50]. Having someone to talk to (another family member and/or professional) may help to address the emotional needs of children [51], as might peer support programs where young people connect with their peers in a fun and supportive environment [52]. Receiving information about mental illness generally and in particular about their parents’ illness may also address children’s anxieties, when delivered in a child focused manner [53].

This study sought to synthesize research in regard to family experiences when a parent is hospitalized for their mental illness. Though further research is necessary to confirm and extend these findings, the review highlighted the importance of models of care that include family friendly visiting rooms and appointment times, but also competent staff and procedures to support the parent, children, and other family members during this time. Such efforts will deliver benefits not only the parent but also to his or her family, especially children.

## Data Availability

All citations identified are in the public domain. The datasets used during the current study are available from the corresponding author on reasonable request.

## Abbreviations

FFP: Family focused practice
PRISMA: The Preferred Reporting Items for Systematic Reviews and Meta-Analyses model
